# TOX‐AD: Exposome Effects of the Environmental Neurotoxicants Lead, Cadmium, and Arsenic on Brain Metal‐Metalloid Accumulation and Alzheimer's Disease Pathobiology

**DOI:** 10.1002/alz70855_106786

**Published:** 2025-12-24

**Authors:** Leandro Ceotto Freitas Lima, Kevin P Kotredes, Juliet A Moncaster, Olga Minaeva, Nicola Lupoli, Gareth R Howell, Paul R Territo, Lee E Goldstein

**Affiliations:** ^1^ Indiana University School of Medicine, Indianapolis, IN, USA; ^2^ The Jackson Laboratory, Bar Harbor, ME, USA; ^3^ Boston University Chobanian & Avedisian School of Medicine, Boston, MA, USA; ^4^ Stark Neurosciences Research Institute, Indiana University School of Medicine, Indianapolis, IN, USA; ^5^ Boston University Alzheimer's Disease Research Center, Boston, MA, USA

## Abstract

**Background:**

While linkage between Alzheimer's disease (AD)/AD‐related dementias (ADRD) and lifetime exposure to environmental toxicants are known, the pathways and mechanisms underpinning them are poorly understood. Variants of specific genes increase AD risk and synergize with lifetime exposures to neurotoxic metals (lead, Pb; cadmium, Cd) and the metalloid (arsenic, As). These metal/metalloid toxicants enter the body (e.g. via contaminated drinking water or food), transit in blood, cross the blood‐brain barrier (BBB), and distribute in the brain, disrupting neuronal function and modulating canonical AD pathways. Metal/metalloid neurotoxicants are suspected modifiers of AD pathobiology. Thus, we hypothesize that Pb, Cd, and As exposures alter the expression of AD‐linked genes, potentiating AD pathogenesis in a toxicant‐specific, neurodevelopment‐sensitive, biomarker‐responsive, and age‐dependent manner. TOX‐AD is a new NIA‐funded consortium project (1U01AG088683) to evaluate this hypothesis and serve as an exposome resource for the AD/ADRD research community.

**Method:**

MODEL‐AD mice B6J.hAPO4.hAβ mice were exposed to Pb (200 ppm), Cd (5, 50 ppm), or As (20 ppm) in drinking water for 30 days vs. controls (unspiked water). B6.APPSwDI mice were also exposed to Pb (13.5, 27, 56 mg/kg) for 1 week to evaluate BBB integrity. Mice were euthanized, blood was collected by cardiac puncture, and tissues were cleared by transcardiac saline perfusion for further analysis.

**Result:**

Animals exposed to environmental toxicants displayed no difference in water intake or daily intake. However, the animals increased levels of metal/metalloids in the blood and brain, altering gene expression associated with increased AD risk, such as ↑VGF (Pb, As) and ↓APP (Cd). B6.APPSwDI animals showed disruption in the BBB integrity, increased Aβ1‐40/Aβ1‐42 ratio, and Aβ deposition. Significant associations for late‐onset AD gene ontology terms: Pb (600 genes): chaperone‐dependent protein refolding (FDR=0.02), glutamate signaling (FDR=0.009), cognition (FDR=0.003), synaptic transmission (FDR=0.01); As (201 genes): synaptic plasticity (FDR=0.02) and organization (FDR=0.04), unfolded protein response (FDR=0.008); Cd (348 genes): protein localization (FDR=0.03), protein metabolism (FDR=0.05), sensory perception (FDR=0.001).

**Conclusion:**

MODEL‐AD mice exposed to Pb, Cd, or As accumulate metal/metalloid toxicants in blood and region‐specific accumulation in brain. Moreover, these exposures revealed toxicant‐specific alterations in gene expression and gene module profiles relevant to AD pathobiology.

